# Assessment of serum inflammatory cytokines in first-episode psychosis: Cross-sectional study

**DOI:** 10.4102/sajpsychiatry.v32i0.2598

**Published:** 2026-04-17

**Authors:** Noxolo Qwabe, Saeeda Paruk, Andrew Tomita, Enver Karim, Usha Chhagan, Vuyokazi Ntlantsana, Bonginkosi Chiliza, Nathlee Abbai, Fazana Dessai, Lindokuhle Thela

**Affiliations:** 1 Discipline of Psychiatry, School of Clinical Medicine, University of KwaZulu-Natal, Durban, South Africa; 2 Centre for Rural Health, School of Nursing and Public Health, University of KwaZulu-Natal, Durban, South Africa; 3 KwaZulu-Natal Research Innovation and Sequencing Platform (KRISP), College of Health Sciences, University of KwaZulu-Natal, Durban, South Africa; 4 Clinical Medicine Laboratory, School of Clinical Medicine, University of KwaZulu-Natal, Durban, South Africa

**Keywords:** immune dysfunction, psychosis, interleukins, serum, first-episode psychosis, schizophrenia, interleukin-6, interleukin-8, interleukin-10, neuroinflammation, inflammation confounders

## Abstract

**Background:**

Chronic low-grade systemic inflammation is implicated in the pathophysiology of schizophrenia, particularly in its early stages. Pro-inflammatory and anti-inflammatory cytokines have been implicated, particularly interleukin (IL) 6, 8 and 10.

**Aim:**

To investigate the association between serum inflammatory cytokines (IL-6, IL-8 and IL-10) and first-episode psychosis (FEP).

**Setting:**

The study was at five clinical sites in KwaZulu-Natal, South Africa.

**Methods:**

This study was an observational, cross-sectional sub-analysis of data derived from a larger longitudinal cohort study of individuals with FEP. In this analysis, we included HIV-negative participants who were 18 years to 45 years old and had less than 6 weeks of antipsychotic exposure. Measures included socio-demographics (age, sex), body mass index (BMI), psychiatric diagnosis (Mini International Neuropsychiatric Interview, MINI), psychosis severity (Positive and Negative Syndrome Scale, PANSS), depressive symptoms (Patient Health Questionnaire-9, PHQ-9), substance use (WHO Alcohol, Smoking and Substance Involvement Screening Test, WHO-ASSIST), childhood trauma (Childhood Trauma Questionnaire, CTQ), and serum cytokines (Multiplex ELISA).

**Results:**

The sample comprised 58 participants, predominantly men (83%), with high rates of lifetime alcohol use (88%) and at least one type of childhood trauma (66%). There was a statistically significantly higher cytokine expression with current alcohol use: IL-6 (*p* = 0.04), IL-8 (*p* = 0.01) and IL-10 (*p* = 0.03). There was also a statistically significant association between lifetime alcohol and tobacco use and elevated IL-10 (*p* = 0.00 and *p* = 0.04, respectively). There was no statistically significant association between PANSS, PHQ-9, CTQ, BMI or cannabis and interleukins.

**Conclusion:**

The study concludes that there is some evidence of immunological dysfunction at baseline in treatment-naïve persons diagnosed with FEP.

**Contribution:**

This study contributes novel data on inflammatory cytokine profiles in individuals with FEP in a South African context, an under-represented population in psychoneuro-immunology research.

## Introduction

The immune system plays a critical role in brain development, influencing synaptic pruning, plasticity and neuronal signalling pathways from early life.^1^ In recent years, increasing evidence has implicated immune dysregulation in the pathophysiology of primary psychotic disorders, including schizophrenia.^2^ This dysregulation is thought to arise from a complex interaction between genetic vulnerability and environmental exposures, such as maternal inflammatory cytokines in utero,^3^ childhood trauma^4^ and substance use.^5^ However, understanding primary psychosis through inflammatory cytokines is limited by inconsistent findings, making it challenging to establish definitive causal links or therapeutic targets.^6,7,8^

Schizophrenia, a severe and chronic psychiatric disorder, is associated with progressive brain changes and neuroinflammation, particularly in the early stages of illness.^9^ Elevated peripheral cytokine levels have been observed both at the onset of psychosis and throughout the illness course.^10^ Among these, specific pro- and anti-inflammatory cytokines have been implicated in symptom severity, cognitive dysfunction and treatment response.^11,12^ These immunological alterations may define a distinct psychosis subtype and offer potential for biomarker-driven intervention.^13^

Three cytokines in particular, interleukin (IL)-6, IL-8 and IL-10, have emerged as promising candidates for understanding immune dysregulation in psychosis.^10,14,15^ IL-6 is a pleiotropic cytokine with both pro- and anti-inflammatory properties, depending on the context and signalling pathway involved.^16^ Notably, IL-6 demonstrates significant diurnal variation in humans, which may affect the interpretation of peripheral measurements.^17^ In first-episode psychosis (FEP), elevated levels of IL-6 and IL-8, both functioning primarily as pro-inflammatory mediators, have been associated with greater severity of positive symptoms, cognitive impairment and poorer treatment response.^10,18,19,20^ Interestingly, lower baseline IL-8 concentrations may also predict subsequent improvements in negative symptoms following treatment,^7^ suggesting a complex, possibly biphasic role for this chemokine in early psychosis. In contrast, IL-10 is anti-inflammatory, and its elevation at psychosis onset has been linked to fewer negative and cognitive symptoms, as well as a more favourable antipsychotic treatment response.^2,15,21^ Studying this balance between pro- and anti-inflammatory cytokines may provide insight into the heterogeneity of early psychosis and inform targeted interventions.

In addition to intrinsic biological factors, environmental exposures such as early-life trauma, substance misuse and obesity contribute significantly to systemic inflammation and may exacerbate psychotic symptoms.^2,4,22^ For instance, childhood adversity has been linked to elevated cytokines in both psychotic and non-psychotic populations,^23,24^ while alcohol and drug misuse are known to activate immune pathways associated with neuroinflammation.^22,25^

Despite the high burden of schizophrenia and psychosis in Africa, few studies have investigated the expression of inflammatory markers in those affected. In South Africa, Sewpaul and colleagues reported elevated C-reactive protein (CRP) levels in individuals with psychological distress although the findings were confounded by multiple variables.^26^ Similarly, Challa et al. reported raised CRP and IL-6 levels among patients with chronic treatment-resistant schizophrenia in Ethiopia.^27^ However, these studies did not focus specifically on individuals with FEP or on a wider panel of relevant cytokines.

Recent extensive reviews, including a systematic review by Halstead et al., have confirmed altered cytokine profiles in psychosis.^28^ However, this study uniquely contributes by exploring cytokine profiles specifically within a human immunodeficiency virus (HIV)-negative South African cohort presenting with FEP, emphasising potential regional and demographic influences.

This study aimed to describe the expression of three key inflammatory cytokines, ILs 6, 8 and 10, in persons diagnosed with FEP, who had received less than 6 weeks of antipsychotic treatment. ILs 6, 8 and 10 were specifically selected as a result of their established roles in inflammatory responses observed in prior schizophrenia studies.^29,30^ IL-6 and IL-8 have demonstrated consistent associations with acute inflammatory responses and structural brain changes in psychosis,^2,14,31^ while IL-10 has shown potential as a modulatory cytokine influencing disease severity and prognosis.^2^

It was hypothesised that elevated levels of IL-6 and IL-8 and lower levels of IL-10 would be associated with greater symptom severity, particularly in the positive and disorganised domains of psychosis. The short treatment duration was selected to minimise the potential confounding anti-inflammatory effects of antipsychotic medications (Mondelli et al.^10^), thus allowing a clearer assessment of baseline immune profiles. This work contributes to the limited body of research from sub-Saharan Africa and may help to clarify early immunological patterns in psychosis.

## Research methods and design

### Study design and parent study

This observational, cross-sectional sub-study forms part of a larger longitudinal investigation, the HIV in FEP study.^32^ The parent study was conducted across multiple psychiatric services in the eThekwini district of KwaZulu-Natal, South Africa, and aimed to examine the clinical, cognitive and immunological characteristics of individuals with FEP, both with and without HIV infection. The present sub-study included only HIV-negative participants and focused specifically on serum inflammatory cytokine expression (IL-6, IL-8 and IL-10). This work forms part of the first author’s Master of Medicine (Psychiatry) dissertation.^33^

Data were analysed from standardised socio-demographic, clinical, psychosocial and laboratory instruments: a structured questionnaire, Mini International Neuropsychiatric Interview (MINI, version 7.0), positive and negative symptom scale (PANSS), Patient Health Questionnaire-9 (PHQ-9), Childhood Trauma Questionnaire (CTQ), WHO-ASSIST and serum cytokine assays using a bead-based multiplex immunoassay. Of note, written informed consent was obtained from all participants before HIV blood collection. Capacity to provide informed consent was assessed clinically, and participants were enrolled in accordance with ethical guidelines for research involving individuals with mental illnesses. The study’s methodology is described in more detail in an article by Chaggan and colleagues.^32^

### Setting

The study was conducted within the public sector providing acute psychiatric services in the eThekwini municipality, KwaZulu-Natal, South Africa. The hospitals involved included Addington Hospital, King Edward VIII Hospital, Prince Mshiyeni Memorial Hospital, RK Khan Hospital and King Dinuzulu Hospital. These hospitals operate in a resource-constrained urban health system with high patient volumes and a substantial burden of severe mental illness, often complicated by comorbid substance use and psychosocial adversity. Psychiatric care is delivered at regional and tertiary levels and forms part of an academic training platform, with services comprising both inpatient units for acute psychiatric stabilisation and outpatient services for follow-up care, reflecting routine clinical practice in the South African public health sector.

### Participants

Eligible participants were adults aged 18 to 45 years presenting with FEP, defined as a first-time DSM-5 diagnosis of a primary psychotic disorder confirmed via the MINI diagnostic interview. Human immunodeficiency virus status was confirmed by fourth-generation ELISA testing, and only HIV-negative participants were included in this sub-study. Additional inclusion criteria were exposure to antipsychotic medication for less than 6 weeks at the time of assessment and the ability to provide informed consent.

Exclusion criteria included antipsychotic exposure exceeding 6 weeks, a prior history of psychosis, concurrent use of corticosteroids or immunosuppressants, any autoimmune condition, uncontrolled chronic medical illness (e.g. epilepsy, neurosyphilis), significant intellectual disability or fewer than 7 years of formal education. Data were collected in English or isiZulu according to participant preference, with a trained bilingual research assistant providing interpretation when required.

Participants were recruited between 03 February 2021 and 20 January 2023. A total of 58 HIV-negative participants had complete clinical and cytokine data at the time of analysis. A minimum sample size of 45 was estimated to ensure 95% confidence with 0.05 precision. As an exploratory study, all available samples were included.

### Data collection

#### Clinical and psychosocial measures

Trained research assistants administered a battery of standardised instruments during participant enrolment. Socio-demographic data were collected by using a structured questionnaire developed for the parent HIV-FEP study and included age, sex, race, educational level, employment status, occupation, residential area, household income, receipt of a disability grant and referral pathway to care. Clinical information included HIV status, duration of untreated psychosis and body mass index (BMI).

Psychiatric diagnoses were confirmed by using the MINI, version 7.0, a structured diagnostic interview aligned with DSM-5 criteria.^33^ It covers a wide range of psychiatric disorders, including psychotic, mood, anxiety, eating and substance use disorders, among others. All questions read must be rated by circling either ‘yes’ or ‘no’ on the right of each question. The time to administer is approximately 15–30 min while maintaining high validity and reliability.^34^ Having been developed as a brief yet accurate interview format that is suitable for both research and non-research clinical settings, its utilisation has been documented in South Africa.^35^

Symptom severity was assessed by using the Positive and Negative Syndrome Scale (PANSS), a 30-item clinician-administered instrument that evaluates positive symptoms, negative symptoms and general psychopathology.^36^ Each item is rated on a seven-point scale, from absent to extreme. The test is known for its validity, reproducibility and reliable factor dimensions.^36^ Each item is rated on a seven-point scale, from absent to extreme. The test is known for its validity, reproducibility and reliable factor dimensions.^36^ The PANSS five-factor model^37^ was used to calculate domain scores for symptom severity. It has been validated for use in individuals with comorbidities and across different diagnostic groups, including in studies conducted in KwaZulu-Natal province, South Africa.^38,39^ This study used the five-factor model comprising positive, negative, disorganised, excitement and depression and/or anxiety subscales, consistent with recommendations for early psychosis.^40,41^ Positive and Negative Syndrome Scale score interpretation followed the classifications proposed by Leucht and colleagues.^42^ Additionally, the total PANSS score was included in the analysis.

Current depressive symptoms were measured using the PHQ-9.^43^ The PHQ-9 is a brief screening tool for depression,^43^ with a sensitivity of 92% and specificity of 80%.^44^ Total scores of 10 and above represent a positive screen for depression. The tool is reliable in screening for depression in persons who are psychotic and has been used and validated in South Africa by several studies.^45,46^

Childhood trauma exposure was assessed by using the Childhood Trauma Questionnaire-short form (CTQ-SF),^47^ a standardised, retrospective 28-item self-report inventory that measures childhood trauma severity and consists of five clinical subscales: emotional, physical and sexual abuse, as well as emotional and physical neglect. Responses are rated on a five-point Likert scale from 1 (‘never’) to 5 (‘very often’), generating scores of 5–25 for each trauma subscale.^47^ Previous studies have demonstrated its validity and reliability, the tool having been used in South Africa.^48,49^

Substance use history was collected by using the World Health Organization Alcohol, Smoking, and Substance Involvement Screening Test (WHO-ASSIST), Version 3.0.^50^ This is an eight-item questionnaire assessing problematic substance use across a person’s lifetime and in the past 3 months.^50^ The assessment includes tobacco, alcohol, cannabis, cocaine, amphetamine-type stimulants (such as ecstasy), inhalants, sedatives, hallucinogens, opioids and ‘other drugs’. Each substance is assigned a risk score, categorised as ‘low risk’, ‘moderate risk’ or ‘high risk’. The risk score guides the recommended intervention, which may be a brief intervention or a brief intervention combined with referral to specialist treatment. The tool has demonstrated reliability and feasibility internationally^51,52^ and has shown similar reliability and feasibility in South Africa.^53^

#### Cytokine measurement and laboratory procedures

Venous blood samples were collected between 08:00 and 12:00 during admission or scheduled outpatient visits. Fasting status was not recorded. Samples were drawn into serum separator tubes. Upon arrival at the Clinical Medicine Laboratory, University of KwaZulu-Natal, the blood was centrifuged at 3000 rpm for 10 min at room temperature, and the serum was stored at −80°C until analysis.

Cytokine concentrations for IL-6, -8, and -10 were measured by using the Bead-Based Multiplex MAP Human Cytokine Assay (Merck, Massachusetts, United States). The samples and controls were run in duplicate using the MAGPIX® multiplexing system (Luminex Corp., Texas, United States), with standard curves being created using the reference standard samples supplied by the manufacturer. Analyte concentrations were calculated using Belysa® Software, the range of the standard curves varying from 3.04 to 9775.04 pg/mL for IL-6, 3.14 to 9639.88 pg/mL for IL-8 and from 2.8 to 9976.08 pg/mL for IL-10.

### Data analysis

Analyses were conducted using complete cases with available clinical and cytokine data, and no imputation of missing data was performed because of the exploratory nature of the study and the modest sample size. All statistical analyses were conducted using STATA version 18. Three types of analyses were performed. Firstly, descriptive statistics were calculated for socio-demographic, clinical and cytokine data. Means and standard deviations were used for continuous variables with normal distribution, while medians and interquartile ranges (IQRs) were reported for skewed distributions. Frequencies and percentages were reported for categorical variables.

Mean and standard deviation were calculated for post-log transformed IL-6, IL-8 and IL-10 cytokine values to obtain a normal distribution.^54^ When normality remained in question, even after log transformation, median and IQRs were reported (as in the case of IL-8, based on the Shapiro-Wilk test).

Secondly, bivariate associations between cytokine levels and socio-demographic or clinical characteristics were assessed. Independent samples t-tests were used for normally distributed log-transformed cytokines (IL-6 and IL-10) and Wilcoxon rank-sum tests for IL-8. Cytokine values were treated as continuous outcome variables in these comparisons.

Thirdly, Pearson’s correlation coefficients were used to assess relationships between log-transformed IL-6 and IL-10 levels and psychotic symptom severity as measured by PANSS domain scores. Spearman’s rank correlation was applied for IL-8. A *p*-value ≤ 0.05 was considered statistically significant.

### Ethical considerations

Ethical clearance to conduct this study was obtained from the University of KwaZulu-Natal (Ref. No.BREC/00004827/2022). The study was conducted by following the South African Department of Health Research Ethics guidelines (2015) and the University of KwaZulu-Natal (UKZN) Policy on Research Ethics. Confidentiality of participant information was strictly maintained.

## Results

### Sample characteristics

The study included 58 participants (Table 1). The majority of participants were men (83%, *n* = 48). Forty per cent (*n* = 23) were classified as overweight. Lifetime tobacco use was reported by 86% (*n* = 50), with the same proportion currently using tobacco (86%, *n* = 50). Lifetime alcohol use was reported by 88% (*n* = 51), and 61% (*n* = 51) reported current alcohol use. Cannabis use was also prevalent, with 84% (*n* = 49) reporting lifetime use and 82% (*n* = 40) reporting current use.

**TABLE 1:** Socio-demographic and clinical characteristics of human immunodeficiency virus–negative participants with first-episode psychosis (*N* = 58).

Variable	*n*	%	Subvariable	*n*	%	Mean	s.d.	Median	IQR
Sex	-	-	Any CTQ	-	-	-	-	-	-
Female	10	17	No	20	34	-	-	-	-
Male	48	83	Yes	38	66	-	-	-	-
BMI	-	-	Emotional abuse	-	-	-	-	-	-
Normal	35	60	No	38	66	-	-	-	-
Overweight	23	40	Yes	20	34	-	-	-	-
Lifetime tobacco	-	-	Physical abuse	-	-	-	-	-	-
No	8	14	No	34	59	-	-	-	-
Yes	50	86	Yes	24	41	-	-	-	-
Current tobacco	-	-	Sexual abuse	-	-	-	-	-	-
No	7	14	No	40	69	-	-	-	-
Yes	43	86	Yes	18	31	-	-	-	-
Lifetime alcohol	-	-	Emotional neglect	-	-	-	-	-	-
No	7	12	No	48	83	-	-	-	-
Yes	51	88	Yes	10	17	-	-	-	-
Current alcohol	-	-	Physical neglect	-	-	-	-	-	-
No	20	39	No	31	53	-	-	-	-
Yes	31	61	Yes	27	47	-	-	-	-
Lifetime cannabis	-	-	PHQ-9	-	-	-	-	-	-
No	9	16	No	37	64	-	-	-	-
Yes	49	84	Yes	21	36	-	-	-	-
Current cannabis	-	-	PANSS	-	-	-	-	-	-
No	9	18	Total score	-	-	84.6	20.2	-	-
Yes	40	82	Negative	-	-	22.2	11.3	-	-
Disorganised	-	-	-	-	-	17.8	7.2	-	-
Positive	-	-	-	-	-	24.6	6.8	-	-
Excitement	-	-	-	-	-	11.6	5.1	-	-
Depression and/or emotional distress	-	-	-	-	-	8.3	3.4	-	-
Log of IL-6	-	-	-	-	-	1.9	1.8	-	-
Log of IL-8	-	-	-	-	-	-	-	3.6	2.4, 4.4
Log of IL-10	-	-	-	-	-	2.5	1.6	-	-

IQR, interquartile range; s.d., standard deviation; CTQ, childhood trauma questionnaire; IL, interleukin; BMI, body mass index; PHQ-9, patient health questionnaire-9; PANSS, positive and negative syndrome scale.

Note: Statistical significance: *p* ≤ 0.05; ‘Yes’ indicate the presence of the condition or positive screen; ‘No’ indicate the absence of the condition or negative screen.

Regarding psychosocial adversity (Table 1), 66% (*n* = 38) of participants reported exposure to at least one form of childhood trauma as measured by the CTQ-short form. Emotional abuse was reported by 34% (*n* = 20), physical abuse by 41% (*n* = 24) and sexual abuse by 31% (*n* = 18). Emotional neglect and physical neglect were reported by 17% (*n* = 10) and 47% (*n* = 27) of participants, respectively. On the PHQ-9, 36% (*n* = 21) screened positive for current depressive symptoms.

### Psychosis severity

Symptom severity was assessed using the PANSS (Table 1). The mean total PANSS score was 84.6 (standard deviation [s.d.] = 20.2). Subscale means were 22.2 (s.d. = 11.3) for negative symptoms, 24.6 (s.d. = 6.8) for positive symptoms and 17.8 (s.d. = 7.2) for disorganised symptoms. The mean score for the excitement subscale was 11.6 (s.d. = 5.1) and for the depression and/or emotional distress subscale, 8.3 (s.d. = 3.4).

### Cytokine concentrations

Peripheral inflammatory markers were assessed using log-transformed values in Table 1. The mean IL-6 concentration was 1.9 (s.d. = 1.8). IL-8 was non-normally distributed even after log transformation (*p*-value of Shapiro-Wilk test was 0.2) and therefore summarised by median and IQR: median = 3.6, IQR = 2.4–4.4. The mean log-transformed IL-10 concentration was 2.5 (s.d. = 1.6).

#### Associations between cytokines and demographic and/or clinical variables

Associations between cytokines and categorical demographic or clinical variables are presented in Table 2. No statistically significant associations were observed between cytokine levels and sex, BMI, tobacco use, cannabis use, CTQ or depressive symptoms (PHQ-9), with all *p*-values > 0.05, except for selected comparisons related to alcohol use.

**TABLE 2:** Association between demographic and clinical variables and serum cytokine levels (log-transformed IL-6, IL-8, and IL-10) among human immunodeficiency virus–negative individuals with first-episode psychosis.

Variable	Group	*n*	Log of IL-6 (*t*-test)				Log of IL-8 (Wilcoxon rank-sum test)					Log of IL-10 (*t*-test)			
			Mean	s.d.	95% CI	*p*-value	Rank sum	Expected	Median	IQR	*p*-value	Mean	s.d.	95% CI	*p*-value
Sex	Female	10	1.87	2.35	0.18; 3.55	0.92	287	295	3.46	3.07	0.87	2.84	1.73	1.40; 4.29	0.56
	Male	48	1.93	1.73	-	-	1424	1416	3.56	1.93	-	2.48	1.60	2.02; 2.95	-
BMI	Normal	35	1.64	1.79	1.03; 2.26	0.16	1031	1032.5	3.32	2.36	0.98	2.32	1.66	1.74; 2.90	0.20
	Overweight	23	2.33	1.85	1.54; 3.14	-	680	678.5	3.64	2.05	-	2.89	1.49	2.22; 3.57	-
Lifetime tobacco	No	8	1.73	1.38	0.58; 2.88	0.76	190	236	2.49	2.26	0.30	1.47	0.70	0.89; 2.05	0.04
	Yes	50	1.90	1.90	1.41; 2.49	-	1521	1475	3.61	1.75	-	2.71	1.65	2.23; 3.20	-
Current tobacco	No	7	1.85	2.28	-0.26; 3.96	0.89	131	178.5	2.98	1.75	0.18	2.87	1.57	1.22; 4.52	0.81
	Yes	43	1.96	1.87	1.39; 2.53	-	1144	1096.5	3.65	2.12	-	2.70	1.68	2.17; 3.23	-
Lifetime alcohol	No	7	2.34	1.57	0.89; 3.79	0.52	242	206.5	4.80	2.48	0.40	0.79	1.34	-0.45; 2.03	< 0.01
	Yes	51	1.86	1.87	1.33; 2.39	-	1469	1504.5	3.53	1.97	-	2.79	1.49	2.36; 3.22	-
Current alcohol	No	20	1.20	1.78	0.37; 2.04	0.04	379	520	3.02	1.54	0.01	2.18	1.38	1.47; 2.89	0.03
	Yes	31	2.29	1.83	1.62; 2.96	-	947	806	3.96	2.74	-	3.13	1.46	2.59; 3.66	-
Lifetime cannabis	No	9	1.65	1.72	0.33; 2.98	0.64	186	265.5	2.61	0.94	0.09	1.76	1.05	0.89; 2.64	0.14
	Yes	49	1.97	1.86	1.43; 2.50	-	1525	1445.5	3.71	1.92	-	2.67	1.66	2.18; 3.15	-
Current cannabis	No	9	1.42	1.94	-0.08; 2.91	0.33	182	225	2.98	1.92	0.27	2.24	1.86	0.53; 3.96	0.47
	Yes	40	2.09	1.85	1.50; 2.68	-	1043	1000	3.76	2.14	-	2.74	1.63	2.22; 3.27	-
Any CTQ	No	20	1.99	1.96	1.07; 2.91	0.83	606	590	3.41	1.87	0.79	2.97	1.50	2.23; 3.72	0.16
	Yes	38	1.88	1.78	1.29; 2.47	-	1105	1121	3.61	2.03	-	2.32	1.63	1.78; 2.87	-
Emotional abuse	No	38	2.03	1.80	1.44; 2.62	0.52	1166	1121	3.61	2.06	0.46	2.61	1.66	2.05; 3.17	0.66
	Yes	20	1.70	1.92	0.80; 2.60	-	545	590	3.2	1.86	-	2.40	1.53	1.67; 3.14	-
Physical abuse	No	34	2.26	1.86	1.62; 2.91	0.09	1056	1003	3.70	2.07	0.40	2.63	1.77	1.99; 3.27	0.61
	Yes	24	1.43	1.72	0.71; 2.15	-	655	708	3.34	1.89	-	2.40	1.37	1.81; 3.00	-
Sexual abuse	No	40	2.02	1.70	1.47; 2.56	0.55	1178	1180	3.58	1.90	0.97	2.50	1.65	1.95; 3.04	0.78
	Yes	18	1.70	2.13	0.64; 2.76	-	533	531	3,39	3.04	-	2.63	1.55	1.83; 3.43	-
Emotional neglect	No	48	1.86	1.83	1.32; 2.39	0.59	1433	1416	3.58	1.96	0.73	2.61	1.60	2.13; 3.09	0.49
	Yes	10	2.20	1.90	0.85; 3.56	-	278	295	3.26	2.35	-	2.21	1.70	1.00; 3.43	-
Physical neglect	No	31	2.11	1.79	1.46; 2.77	0.39	900	914.5	3.28	2.19	0.82	2.79	1.51	2.22; 3.37	0.21
	Yes	27	1.70	1.89	0.95; 2.44	-	811	796.5	3.71	1.75	-	2.25	1.69	1.57; 2.94	-
PHQ-9	No	37	1.98	1.74	1.40; 2.57	0.72	1110	1091.5	3.53	2.36	0.77	2.44	1.71	1.85; 3.03	0.55
	Yes	21	1.80	2.02	0.88; 2.72	-	601	619.5	3.59	1.62	-	2.71	1.43	2.04; 3.38	-

IL, interleukin; BMI, body mass index; CTQ, childhood trauma questionnaire; PHQ-9, patient health questionnaire-9; s.d., standard deviation; CI, confidence interval; IQR, interquartile range.

Note: Comparisons for IL-6 and IL-10 were conducted using independent samples *t*-tests. IL-8 was analysed using the Wilcoxon rank-sum test as a result of non-normal distribution. ‘Yes’ indicate the presence of the condition or positive screening result. ‘No’ indicate the absence of the condition or negative screening result. Statistical significance: *p* ≤ 0.05.

Current alcohol use was associated with significantly higher mean levels of IL-6 (*p* = 0.04), IL-8 (*p* = 0.01) and IL-10 (*p* = 0.03). Lifetime alcohol use was also associated with higher IL-10 levels (*p* ≤ 0.01). Additionally, lifetime tobacco use was associated with significantly higher IL-10 levels (*p* = 0.04).

#### Associations between positive and negative symptom scale scores and cytokines

Table 3 presents the associations between PANSS domain scores and log-transformed cytokine levels. Associations were assessed by using Pearson’s correlation coefficients. None of the associations met the threshold for statistical significance (*p* ≤ 0.05). The strongest observed associations, although not statistically significant, included a positive correlation between IL-10 and positive symptom scores (*β* = 0.25, *p* = 0.06) and between IL-8 and disorganised symptom scores (*β* = 0.23, *p* = 0.08). All other associations were small in magnitude, with *p*-values ranging from 0.12 to 0.94.

**TABLE 3:** Association between positive and negative syndrome scale domain scores and log-transformed cytokine levels (IL-6, IL-8 and IL-10) in human immunodeficiency virus-negative individuals with first-episode psychosis.

PANSS scores	IL-6	IL-6: *p*-value	IL-8	IL-8: *p*-value	IL-10	IL-10: *p*-value
Total score, mean (s.d.)	0.10	0.45	0.04	0.78	0.08	0.58
Negative, mean (s.d.)	−0.02	0.89	−0.01	0.94	−0.09	0.50
Disorganised, mean (s.d.)	0.10	0.48	0.23	0.08	−0.07	0.60
Positive, mean (s.d.)	0.17	0.21	−0.08	0.57	0.25	0.06
Excitement, mean (s.d.)	0.11	0.43	0.07	0.63	0.21	0.12
Depression and/or emotional distress	−0.03	0.83	−0.18	0.17	0.09	0.53

PANSS, positive and negative syndrome scale; IL, interleukin; s.d., standard deviation.

Note: Associations were evaluated using linear regression models. Reported values are unstandardised beta coefficients and corresponding *p*-values for each symptom domain. Statistical significance: *p* ≤ 0.05.

## Discussion

This observational study examined the associations between serum inflammatory cytokines (IL-6, IL-8 and IL-10) and clinical, psychosocial and demographic variables in HIV-negative individuals with FEP. There was no association between ILs and the socio-demographic variables, such as gender and age, consistent with inconsistent findings in previous studies that investigated these associations.^15,27,55^

Aligning with the global literature, our study parallels past investigations but presents variations. While international studies such as Garcia-Rizo et al. (2011) and Miller et al.^30^ reported elevated IL-6 levels in early psychosis, our findings do not show significant associations. Contrary to expectations and prior findings from several international studies, we did not observe statistically significant associations between log-transformed serum concentrations of IL-6, IL-8 or IL-10 and psychosis symptom domains as assessed by the PANSS.^8,11,56^ Conflicting outcomes, as seen in studies by Bocchio-Chiavetto et al. and He et al., highlight inconsistencies across different psychosis stages and subtypes, underscoring the need for more comprehensive investigations utilising well-defined cohorts to delineate the specific roles of IL-6 and IL-8 in psychosis.^6,7^ Several factors may account for the lack of observed associations. Firstly, cytokines were measured in the serum rather than in cerebrospinal fluid (CSF), and levels of serum cytokines may not reflect neuroinflammation occurring centrally in the brain, which is a key site of pathology in schizophrenia^9,57,58^ Secondly, previous meta-analyses have highlighted methodological limitations across cytokine studies, including heterogeneous participant profiles, varied antipsychotic exposure and differences in laboratory techniques.^2,30^ Thirdly, not all presentations of schizophrenia may be driven by inflammation. Schizophrenia is a polygenic and heterogeneous disorder, and only a subset of patients may exhibit immune dysregulation.^59^ Genetic vulnerability may further modulate inflammatory responses, as polymorphisms within immune-related genes, particularly those within the human leukocyte antigen (HLA) region, have been associated with altered cytokine expression and schizophrenia risk.^59,60^ In addition, environmental and behavioural confounders such as substance use, metabolic status, psychosocial stress, diet and co-infections can substantially influence peripheral cytokine levels and may obscure associations with psychopathology when not fully controlled.^2,25,26^

It has been suggested that symptom clusters, such as negative symptoms, are more consistently associated with elevated cytokine levels.^9,13^ However, in our study, even when using a five-factor PANSS model, no significant associations emerged between cytokine levels and disorganised, negative or depressive symptom dimensions. Our cohort was intentionally limited to individuals who had been exposed to antipsychotics for fewer than 6 weeks, but future studies may benefit from tighter stratification based on inflammatory subtypes or clinical phenotypes.

Of interest was the absence of a discernible link between IL-6 and psychosis symptoms, despite our participants having a high mean PANSS score of 84.6. This finding contrasts with the common perception that IL-6 is frequently identified as the most dysregulated IL in psychosis patients, as indicated by a meta-analysis and multiple other studies.^18,19,29,30^ The meta-analysis specifically reported correlations between IL-6, treatment response and metabolic outcomes, which were not evident in our cohort, possibly as a result of the different patient profiles, such as poor socio-economic circumstances, in this study. However, several other studies were consistent with our result and found no significant differences in IL-6, IL-8 and IL-10 in first-episode schizophrenia.^61^ Reflecting on our findings, the inclusion of a control arm may have provided a more comprehensive assessment of the significance of peripheral inflammation in FEP.

While stratifying study participants based on the severity of negative symptoms could address this issue, as these symptom clusters appear to be associated with interleukins,^9,13^ our results did not find any association between interleukins and individual PANSS subscales. Several studies have found no significant differences in IL-6, IL-8 and IL-10 during acute episodes of schizophrenia or a link between IL-8 and overt schizophrenia psychopathology.^4,56^ Speculation by various authors suggests that older participants and chronically ill patients might present with higher peripheral inflammation.

Despite the absence of significant associations between serum IL-6, IL-8 and IL-10 levels and psychosis symptom severity in this sample of HIV-negative individuals with FEP, our study yielded important findings regarding the role of substance use in modulating peripheral inflammatory markers. Specifically, current alcohol use was significantly associated with elevated IL-6 (*p* = 0.04), IL-8 (*p* = 0.01) and IL-10 (*p* = 0.03), while lifetime alcohol use and tobacco use were also significantly associated with higher IL-10 levels. These associations are consistent with previous studies showing that alcohol and nicotine exposure induce systemic inflammation through mechanisms such as increased gut permeability, oxidative stress and activation of microglia.^24^ The strength and consistency of these findings suggest that substance use may act as a key environmental modulator of cytokine expression in individuals with early psychosis, and failure to account for such confounders may explain inconsistencies in the literature. While we observed no significant associations between ILs and cannabis use, this failure may reflect limited power to detect subtle effects or the complex immunomodulatory role of cannabinoids. Regarding inflammation in schizophrenia, studies have shown that individuals with abnormal inflammatory cytokine expression tend to have a genetic predisposition. There is evidence that individuals with psychosis and inflammation have genetic polymorphisms of the HLA gene^60^; this genetic predisposition was not being explored in this study.

The observed link between inflammatory markers and substance use, but not psychopathology, underscores the need to consider modifiable environmental exposures when interpreting cytokine findings. These results support the view that elevated inflammatory markers in FEP may sometimes be secondary to behavioural or lifestyle factors rather than psychosis per se.^2,30^

Regarding childhood trauma, our findings showed no significant associations with IL-6, IL-8 or IL-10, diverging from earlier work linking early adversity to elevated cytokines in psychosis.^4,23^ However, our sample was restricted to FEP patients, whereas many prior studies included individuals with chronic illness, which may have amplified cumulative inflammatory effects.^7,20^

The absence of neuroimaging data in this study is a limitation. Furthermore, cytokine levels in serum may fluctuate because of diurnal variation, metabolic status and psychosocial stress, which were not systematically controlled.^62^

Finally, while previous meta-analyses^30^ and early-stage studies^18^ report associations between IL-6 and poor cognitive and treatment outcomes, we did not find support for such a relationship in our sample. This discrepancy may be a result of population differences, including high levels of socio-economic adversity, undernutrition or possible unmeasured co-infections that are more prevalent in low- and middle-income countries.^26,27^

The absence of a healthy control group in our study limits the interpretability of cytokine levels, as does the reliance on peripheral (rather than central) markers. Nevertheless, our study contributes valuable data from a high-burden, under-researched setting, using a clearly defined FEP cohort with minimal antipsychotic exposure, a key methodological strength.

### Strengths and limitations

This study is among the few to examine serum inflammatory cytokines in a South African cohort of HIV-negative individuals with FEP. Its strengths include the use of validated psychometric tools, standardised cytokine assays and strict antipsychotic exposure limits to minimise pharmacological confounding. However, several limitations must be acknowledged. Importantly, the study relied exclusively on peripheral (serum) cytokine measurements, which may not accurately reflect central nervous system inflammatory processes, as peripheral and central immune markers do not consistently correlate in psychosis. Future studies would benefit from integrating cerebrospinal fluid biomarkers and neuroimaging measures of neuroinflammation to better characterise central immune mechanisms. The absence of a healthy control group limits direct comparison with non-psychotic populations, and the modest sample size may have reduced power to detect smaller effect sizes. Although participants with known inflammatory or autoimmune conditions were excluded, residual confounding from unmeasured factors such as diet, metabolic status or undetected co-infections remains possible. Trauma exposure was assessed using brief screening tools rather than comprehensive batteries, potentially limiting sensitivity. Finally, the cross-sectional design precludes causal inferences regarding the temporal relationship between immune dysregulation and psychosis.

## Conclusion

This study did not find a significant association between serum inflammatory cytokines and psychosis symptom domains in individuals with FEP. However, clear associations between alcohol and tobacco use and elevated cytokine levels, particularly IL-10, highlight the relevance of modifiable environmental factors in interpreting immune biomarkers in psychosis. These findings add to the growing evidence that inflammatory processes in psychosis are heterogeneous and context dependent, rather than uniformly linked to symptom severity. Future research should prioritise larger, longitudinal cohort studies that integrate genetic profiling, neuroimaging markers of neuroinflammation and repeated peripheral and cerebrospinal fluid immune assessments, alongside comprehensive clinical phenotyping and appropriate control groups, to better delineate inflammatory subtypes and clarify the temporal relationship between immune dysregulation and the onset and progression of psychosis.
